# La plateforme de lomé : un plaidoyer porté par le mouvement mutualiste

**DOI:** 10.48327/mtsimagazine.n1.2021.80

**Published:** 2021-04-02

**Authors:** T. Kanga-Tona

**Affiliations:** Association Internationale de la Mutualité, Rue d'Arlon 50 B-1000 Brussels, Belgique

**Keywords:** Mutuelles, Couverture santé universelle, Assurance santé, Protection sociale, Afrique, Coopération, Objectifs de développement durable, ODD, Mutual insurances, Universal health coverage, Health insurance, Social protection, Africa, Cooperation, Sustainable Development Goals, SDGs

## Abstract

L'adoption de la Plateforme d'Abidjan en 1999 a accompagné l'essor des mutuelles de santé sur le continent africain. Vingt ans après l'adoption de la Plateforme d'Abidjan, les mutuelles de santé ont effectivement connu un développement, une structuration ainsi qu'une professionnalisation certains. En janvier 2019, le mouvement mutualiste international a adopté au cours de la conférence de Lomé la « Plateforme de Lomé », un document présentant un nouveau consensus de développement de la mutualité, prenant en compte l'impact des niveaux de décisions nationaux, régionaux et internationaux sur l'environnement influençant le développement des mutuelles de santé. Deux ans après l'adoption du document, ses préconisations restent pertinentes et la crise de la COVID-19 révèle l'importance qu'il y a à se saisir de toutes les ressources afin de développer les systèmes de santé sur le continent africain.

## Abstract (en)

The adoption of the Abidjan Platform in 1999 accompanied the development of mutual health insurance on the African continent. Twenty years after the adoption of the Abidjan Platform, mutual health insurance companies have indeed experienced development, structuring and professionalization. In January 2019, the international mutualist movement adopted the “Lomé Platform” during the Lomé conference, a document presenting a new consensus for the development of mutuality, taking into account the impact of national, regional and international decision-making levels on the environment influencing the development of mutual health insurance. Two years after the adoption of the document, its recommendations remain relevant and the COVID-19 crisis reveals the importance of seizing all resources to develop health systems on the African continent.

La Plateforme de Lomé^1^ est le résultat d'un processus de réflexion et de rédaction collectives des différents acteurs concernés par la promotion des mutuelles mené, à la fois, à partir de l'Afrique et de l'Europe. La Plateforme a été adoptée à l'issue de la Conférence de Lomé qui s'est déroulée le 22 et 23 janvier 2019 à Lomé sous le Haut-Patronage de la Présidence de la République togolaise et en collaboration avec l'Union Economique et Monétaire Ouest-Africaine (UEMOA). La conférence a été l'occasion de faire le bilan du développement du mouvement mutualiste africain au cours des vingt dernières années précédant la conférence (1998 – 2018), des éléments de succès, ainsi que de poser les jalons pour continuer le développement du mouvement mutualiste dans le futur. La conférence s'est inscrite dans la poursuite de l'engagement politique en faveur de la reconnaissance et du développement du mouvement mutualiste.

La Plateforme de Lomé est un document porté par le mouvement mutualiste africain et soutenu par le monde mutualiste international. Elle reprend des propositions et des revendications qui sont adressées aux organisations supranationales et aux décideurs politiques des différents pays africains. Le succès enregistré par le mouvement mutualiste au cours des vingt dernières années se place dans un contexte international spécifique. L'Organisation des Nations Unies (ONU) travaille afin de remplir ses objectifs de développement durable dont deux portent sur l’éradication de la pauvreté ainsi que sur la santé et le bien-être. L'Organisation Internationale du Travail (OIT) a fêté ses cent ans en 2019, alors qu'elle a également adopté des recommandations sur la protection sociale. L'Union Africaine (UA) a également adopté l'Agenda 2063, posant un cadre ambitieux pour le développement du continent pour les cinquante prochaines années. Le Directeur Général de l'Organisation Mondiale de la Santé (OMS) a plusieurs fois rappelé son ambition de donner l'accès universel aux soins de santé à un milliard de personnes en plus d'ici 2030.

C'est dans ce contexte que les mutuelles, à travers la Plateforme de Lomé prônent trois axes d'action. Ces axes sont à la fois une feuille de route à suivre pour le mouvement mutualiste, ainsi que des revendications à porter au niveau des décideurs politiques nationaux. Il revient désormais aux organisations internationales de favoriser le développement du mouvement mutualiste au niveau national en s'inspirant de ces trois axes.

Au rang de l'engagement politique, le mouvement mutualiste demande le renforcement ou la mise en œuvre de dispositifs légaux régissant les mutuelles de santé. Ces dispositifs doivent être mis en place par des mesures d'application, garantissant ainsi la viabilité des mutuelles des points de vue institutionnel, technique, financier et fonctionnel. L'existence d'un environnement légal présentant des droits opposables ainsi que des obligations incombant aux mutuelles est le premier élément pour aider au développement de celles-ci. Cela favorisera leur contribution à l'atteinte des objectifs de la couverture de santé universelle, selon les critères fixés par les gouvernements et dans l'optique de satisfaire les standards internationaux de développement.

Au rang de l'adhésion obligatoire, le mouvement mutualiste demande l'instauration et la mise en œuvre effective de mécanismes d'adhésion obligatoire à la couverture sanitaire pour l'ensemble de la population, reposant sur un processus évolutif, allant de la conception, aux phases de transition et à l'implémentation. Seul un système d'assurance obligatoire est en mesure d'assurer la solidarité, la mutualisation des risques, ainsi que la pérennité des ressources. Ce sont trois caractéristiques clés des systèmes de protection de santé efficaces. L'adhésion obligatoire peut être assortie de mesures incitatives comme le conditionnement de l'accès à certains documents à la souscription à la couverture santé nationale, à intégrer dans un cadre légal.

Au rang de la délégation de gestion, le mouvement mutualiste demande la gestion et l'organisation de la couverture de santé universelle reposant sur une délégation de gestion confiée aux mutuelles par l’État. Les spécificités inhérentes au modèle mutualiste au rang desquelles la fiabilité, la proximité, la démarche participative, la défense croisée de l'intérêt des citoyens et de la cohérence du système de protection sociale, sont des avantages. Ces caractéristiques en font des parties-prenantes incontournables.

Pour la majorité des pays africains, les dépenses de santé sont largement en deçà de ce qui est recommandé. L'Organisation de Coopération et de Développement Economique (OCDE) estime qu'en 2014 un tiers des pays africains avaient des dépenses de santé par habitant en dessous du seuil acceptable, fixé à 38 USD courant^2^. Le financement de la santé dans ces pays est habituellement effectué par les programmes de santé d'organismes internationaux ou d'associations humanitaires. En l'absence de ces derniers, les dépenses de santé sont financées par la population au travers des paiements directs qui représentent environ 90 % des dépenses de santé totales. Ces paiements directs représentent pour la majorité des ménages des dépenses catastrophiques, conduisant généralement à un renoncement aux soins.

La protection sociale et la couverture des soins de santé sont donc des points d'attention pour le futur. C'est le message que l'AIM a véhiculé dans le cadre de la conception et de l'adoption du partenariat Union européenne – Afrique. Une occasion de rappeler que toute politique de soutien au développement ne peut faire l’économie du renforcement des systèmes de santé dans les pays où cette dernière se déploie. La crise du coronavirus, ses effets dévastateurs sur les économies du monde entier a mis en avant l'importance de la constitution de systèmes de santé forts, fiables, résilients et dûment financés. Le mouvement mutualiste africain et moyen-oriental a déjà adopté une déclaration^3^ sur la riposte à la COVID-19 ainsi que sur la préparation post-COVID-19, s'appuyant sur les réalités des sociétés en Afrique et au Moyen-Orient en essayant de dessiner un chemin authentiquement africain et mutualiste de sortie de crise et de développement socioéconomique, qui demande à:

1. S'appuyer sur les mutuelles et la société civile pour assurer une réponse inclusive à la pandémie et pour œuvrer à la protection sociale-santé universelle
2. Renforcer et équiper les systèmes de santé et de protection sociale
3. Répondre à la crise socio-économique, le réel défi qui attend l'Afrique

Le mouvement mutualiste a pris ses responsabilités durant la crise du coronavirus. Les enseignements tirés de la crise ainsi que ses conséquences sur le contenu de la Plateforme de Lomé ne manqueront pas d’être à l'agenda d'une prochaine conférence internationale mutualiste. La date de l’évènement reste encore à déterminer.

## Conflits d'intérêts

Les auteurs ne déclarent aucun conflit d'intérêts.

